# Supplementary material for: Extracellular Vesicles Inhibit the Response of Pancreatic Ductal Adenocarcinoma Cells to Gemcitabine and TRAIL Treatment
Source: Int J Mol Sci. 2022 Jul 15;23(14):7810. doi: 10.3390/ijms23147810 (PMC9317709; doi:10.3390/ijms23147810)
Supplement: Supplementary file 1 [file ijms-23-07810-s001.zip › ijms-1754126-Supplementary.pdf]

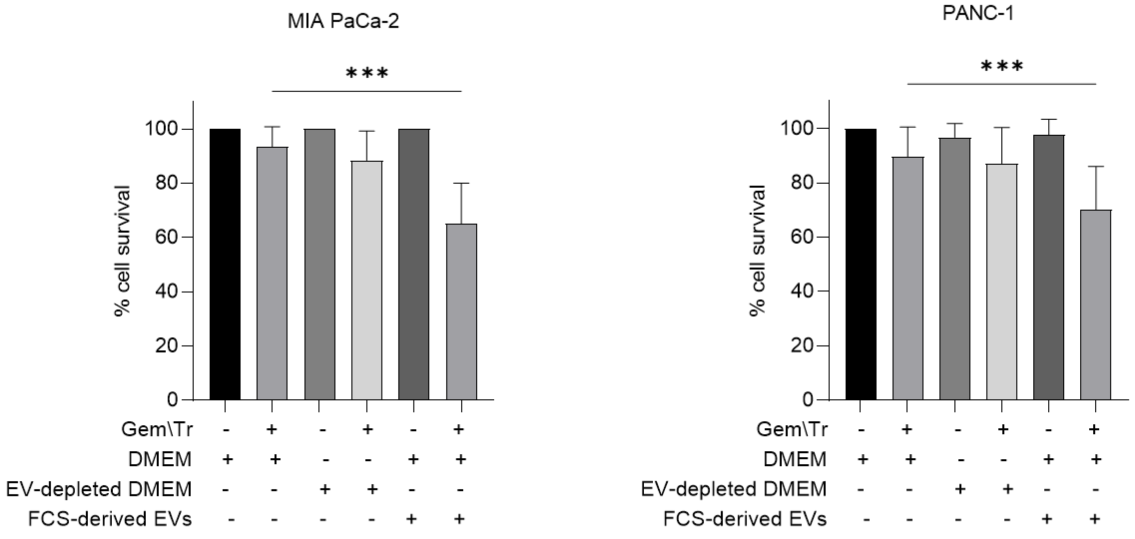


(**a**) (**b**)

**Supplementary Figure S1.** Effect of EV-depleted DMEM and FCS-derived EVs on response of PDAC cells to gemcitabine and TRAIL treatment. MIA PaCa-2 and PANC-1 cells were seeded in 96-well plates at 3 x $10^4$ cells/$cm^2$. The sensitivity of cells to the combination treatment was assessed using MTT assays. **a**) MIA PaCa-2, and **b**) PANC-1 cells were treated with 100 μM gemcitabine for 24 h and 100 ng/ml TRAIL for the final 4 (for MIA PaCa-2) or 6 h (for PANC-1) of the gemcitabine treatment. EV-depleted DMEM and FCS-derived EVs were added for the duration of TRAIL treatment. Data are provided as means ± SD. Student's t-test was used to determine statistical significance between gemcitabine and TRAIL alone and in combination with FCS-derived EVs: P *** P<0.001.

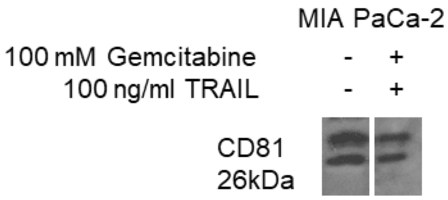


**Supplementary Figure S2.** Expression of EV marker CD81 in MIA PaCa-2 cells**.** MIA PaCa-2 cells were used to condition medium following treatment with gemcitabine for 24 h and 100 ng/ml TRAIL for the final 4h. This medium was subject to differential ultracentrifugation, and the resulting pellet was lysed using supplemented RIPA buffer. Extracts were subject to SDS-PAGE and transferred to PVDF membranes, before probing for CD81. EV – extracellular vesicle; CD81 – cluster of differentiation; RIPA buffer – radioimmunoprecipitation buffer.

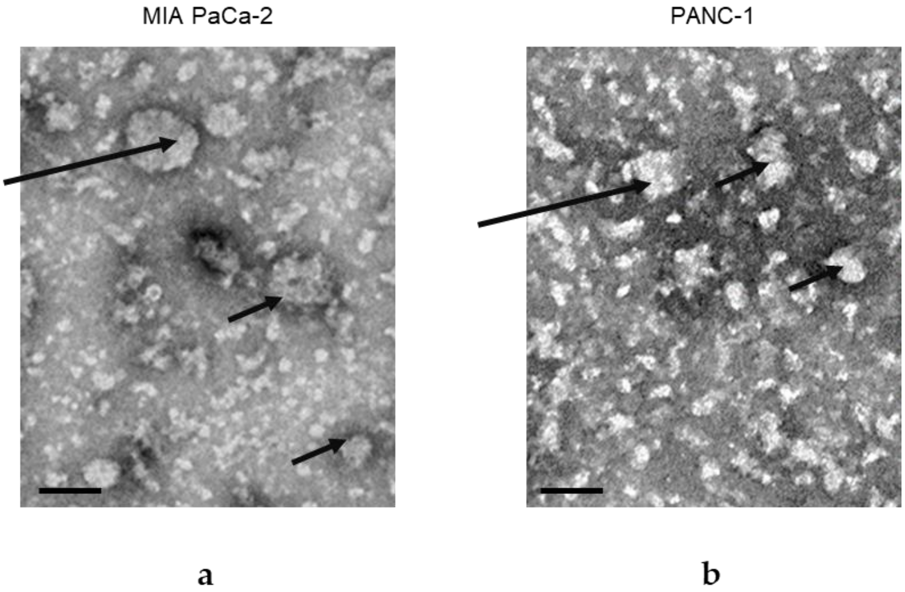


**Supplementary Figure S3.** Transmission Electron Microscopy Images of EVs from MIA PaCa-2 and PANC-1 cell lines. Conditioned media taken from MIA PaCa-2 and PANC-1 cells was used to purify EVs using differential centrifugation as described in material and methods. Transmission electron microscopy (TEM) showing EV morphology – see arrows pointing at EVs (scale bar is indicated at 50 nm). EV – extracellular vesicles; TEM – transmission electron microscopy.
